# Reduction in COVID-19 Patients Requiring Mechanical Ventilation Following Implementation of a National COVID-19 Vaccination Program — Israel, December 2020–February 2021

**DOI:** 10.15585/mmwr.mm7009e3

**Published:** 2021-03-05

**Authors:** Ehud Rinott, Ilan Youngster, Yair E. Lewis

**Affiliations:** ^1^Department of Public Health, Faculty of Health Sciences, Ben-Gurion University of the Negev, Beer-Sheva, Israel; ^2^Sackler School of Medicine, Tel-Aviv University, Tel-Aviv, Israel; ^3^Maccabi Healthcare Services, Tel-Aviv, Israel.

The availability of COVID-19 vaccines represents an opportunity to mitigate the effects of the global pandemic. Achieving high vaccination coverage through intensive vaccination campaigns has the potential to substantially reduce COVID-19–associated morbidity and mortality. Clinical trials have demonstrated the efficacy of COVID-19 vaccines in preventing mild and severe COVID-19 in a controlled setting. However, clinical trials are not designed to assess the population impact of vaccination in a real-world setting (*1*,*2*). Israel initiated a national vaccination campaign using the Pfizer-BioNTech BNT162b2 (Pfizer-BioNTech) vaccine in December 2020, prioritizing persons aged >60 years, health care workers, and persons with underlying medical conditions. By February 2021, 2-dose vaccination coverage among persons aged ≥70 years was 84%. To assess the effect of COVID-19 vaccination on the occurrence of severe disease, an ecological study was conducted. Requiring mechanical ventilation was used as a proxy for severe COVID-19. The number of COVID-19 patients aged ≥70 years (who had the highest 2-dose vaccination coverage, 84.3%) requiring mechanical ventilation was compared with that of patients aged <50 years, who had the lowest 2-dose vaccination coverage (9.9%). Since implementation of the second dose of the vaccination campaign, the ratio of COVID-19 patients requiring mechanical ventilation aged ≥70 years to those aged <50 years has declined 67%, from 5.8:1 during October–December 2020 to 1.9:1 in February 2021. These findings provide preliminary evidence of the effectiveness of vaccines in preventing severe cases of COVID-19 at the national level in Israel. Receipt of COVID-19 vaccines by eligible persons can help limit spread of disease and potentially reduce the occurrence of severe disease.

The first case of COVID-19 in Israel, a country with a population of approximately 9 million, was reported in February 2020. As of February 9, 2021, approximately 700,000 cases and 5,200 deaths had been reported (*3*). Nonpharmaceutical interventions have included three national stay-at-home orders,^†^ multiple rounds of school closures, restrictions on commercial activity and travel, and a mask mandate, among others. The most recent stay-at-home order was implemented on January 8, 2021, amid a nationwide surge in cases (*4*). On December 20, 2020, Israel initiated a national vaccination program against COVID-19, using the Pfizer-BioNTech vaccine and prioritizing persons aged ≥60 years, health care workers, and persons with chronic conditions that increase risk for infection or severe disease (*5*).

To assess the impact of COVID-19 vaccination on the occurrence of severe COVID-19 at the population level an ecological study was conducted using the number of COVID-19 patients requiring mechanical ventilation as a proxy for severe disease. The number of COVID-19 patients requiring mechanical ventilation aged ≥70 years, who had the highest 2-dose COVID-19 vaccination coverage, was compared with the number of those aged <50 years, who had the lowest 2-dose coverage. COVID-19 vaccine administration data during December 20, 2020–February 9, 2021, were obtained from publicly available Israel Ministry of Health data (*6*). Vaccinated persons with missing age data were excluded from the analysis. Daily numbers of COVID-19 patients receiving mechanical ventilation between October 2, 2020, and February 9, 2021, (including during the second and third stay-at-home orders) were obtained from the Israel Ministry of Health COVID-19 dashboard using a publicly available repository.^§^ Vaccination status is not available for individual patients in this repository. Population data were drawn from the Israel Central Bureau of Statistics as of the end of 2019.

By February 9, 2021, a total of 3,606,858 persons had received the first vaccine dose, and among those, 2,223,176 (62%) had received the second dose. Two-dose COVID-19 vaccination coverage among persons aged ≥70 years, 60–69 years, 50–59 years, and <50 years was 84.3%, 69.0%, 50.2%, and 9.9%, respectively (Figure 1).

**FIGURE 1:**
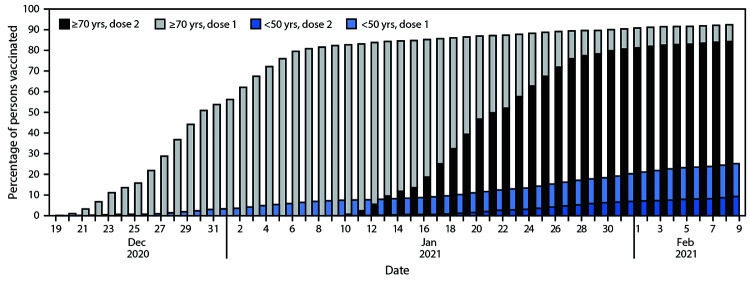
First- and second-dose COVID-19 vaccination coverage* among persons aged <50 and ≥70 years† — Israel, December 20, 2020–February 9, 2021 * Dose 2 shaded areas include those who received dose 1. ^†^ Total population: 6.4 million (aged <50 years); 0.735 million (aged ≥70 years).

During October 2, 2020–February 9, 2021, the median daily numbers of COVID-19 patients aged <50 years and ≥70 years who required mechanical ventilation were 15 (range = 6–63) and 84 (range = 45–127), respectively. During October 8–December 30, 2020, the mean ratio of ventilated patients aged ≥70 years to those aged <50 years was 5.8:1 (99% confidence interval = 5.5–6.1; range = 4.2–8.5). During the last week of January 2021, although the average daily number of ventilated patients aged ≥70 years had begun to decline, the average daily number of ventilated patients aged <50 years was still increasing (Figure 2). By February 9, 2021, the 7-day rolling average number of ventilated patients aged ≥70 years was 109, and among those aged <50 years was 57.7 (ratio = 1.9:1), representing a 67% decrease in the ratio compared with that during October 8–December 30, 2020.

**FIGURE 2:**
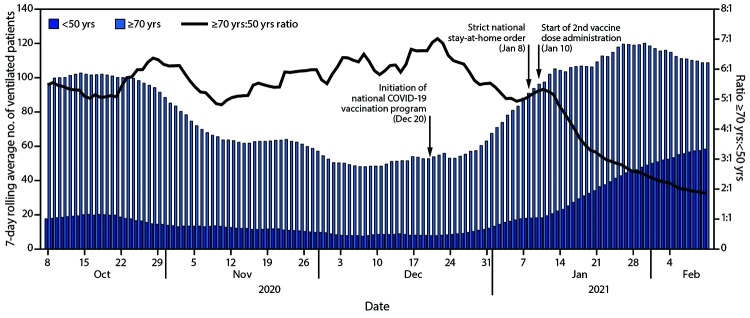
Number and ratio of COVID-19 patients aged <50 and ≥70 years requiring mechanical ventilation — Israel, October 8, 2020–February 9, 2021

## Discussion

These findings suggest a possible impact of the nationwide COVID-19 vaccination campaign in Israel on reducing severe COVID-19 requiring mechanical ventilation. The Israeli national vaccination campaign (*5*), which was initiated on December 20, 2020, in the midst of a nationwide surge of COVID-19 cases, was followed by a strict national stay-at-home order starting on January 8, 2021 (*4*). Vaccine rollout was rapid, and because older age groups were prioritized for vaccination (*5*), it was feasible to compare the number of patients requiring mechanical ventilation between the oldest and youngest age groups, whose COVID-19 vaccination coverage rates differed the most. The percentage of COVID-19 patients aged ≥70 years requiring mechanical ventilation in Israel fluctuated during October–December 2020 but has considerably and consistently decreased after implementation of the vaccination campaign prioritizing older adults. The decline in the ratio of persons aged ≥70 years to those aged <50 years requiring mechanical ventilation began around the time of commencement of administration of the second vaccine dose (January 10, 2021). This might reflect the effects of the first dose, an observation that is consistent with the Pfizer-BioNTech vaccine phase 3 results, which demonstrated partial efficacy after the first dose (*1*).

Considering the vaccination rate and the expected vaccine efficacy, this study provides preliminary evidence at the population level for the reduction in risk for severe COVID-19, as manifested by need for mechanical ventilation, after vaccination with the Pfizer-BioNTech COVID-19 vaccine. These data are consistent with preliminary reports showing a reduction in COVID-19 cases and severe cases in the vaccinated population and a reduction in viral load in vaccinated persons compared with that in unvaccinated persons.^¶^^,^**^,^^††^ Taken together, these results suggest reduced rates of severe COVID-19 following vaccination.

The findings in this report are subject to at least three limitations. First, this was an ecological analysis that relied on preliminary and aggregated data and might be subject to delays in reporting of COVID-19 cases. Second, the longitudinal and observational nature of this study limited the ability to account for different concomitant effects, including development and spread of novel variants, the general increase in COVID-19 cases and national stay-at-home orders. However, by analyzing the percentage of cases by age group (accounting for vaccination rates), these results are unlikely to be influenced by the overall incidence in the population. Finally, there were possible differences in adherence to mitigation measures between the age groups. To address this limitation, the analysis period was extended to include an earlier period with a stay-at-home order (September–October 2020).

Many countries are currently conducting national COVID-19 vaccine campaigns. The findings from this study provide preliminary but important evidence of the effectiveness of vaccines in preventing severe cases of COVID-19 at the national level in Israel. Receipt of COVID-19 vaccines by eligible persons can help limit spread of disease and potentially reduce the occurrence of severe disease.

**Summary**

**What is already known about this topic?**

Clinical trials have demonstrated the efficacy of COVID-19 vaccines in a controlled setting. Israel initiated a national vaccination campaign in December 2020, prioritizing persons aged >60 years and other high-risk populations.

**What is added by this report?**

By February 2021, 2-dose vaccination coverage was 84% among persons aged ≥70 years and 10% among those aged <50 years. The ratio of COVID-19 patients aged ≥70 years requiring mechanical ventilation to those aged <50 years declined 67% from October–December 2020 to February 2021.

**What are the implications for public health practice?**

These findings provide preliminary evidence of the effectiveness of vaccines in preventing severe cases of COVID-19 at the national level in Israel.

## Footnotes

* These authors contributed equally to this report.

† The dates of the three stay-at-home orders were March 12–April 19, 2020; September 18–October 18, 2020; and January 8–February 7, 2021.

§
https://github.com/dancarmoz/israel_moh_covid_dashboard_data

¶
https://www.medrxiv.org/content/10.1101/2021.02.06.21251283v1

**
https://www.medrxiv.org/content/10.1101/2021.02.08.21251325v1

††
https://www.medrxiv.org/content/10.1101/2021.02.02.21250630v1
